# Facile Solution-Refluxing Synthesis and Photocatalytic Dye Degradation of a Dynamic Covalent Organic Framework

**DOI:** 10.3390/molecules27228002

**Published:** 2022-11-18

**Authors:** Xiao-Lian Wang, Yi-Ying Sun, Yonghong Xiao, Xiao-Xian Chen, Xiao-Chun Huang, Hao-Long Zhou

**Affiliations:** 1 Department of Chemistry and Key Laboratory for Preparation and Application of Ordered Structural Materials of Guangdong Province, Shantou University, Shantou 515063, China; 2 Chemistry and Chemical Engineering Guangdong Laboratory, Shantou 515031, China; 3 Key Laboratory of Organosilicon Chemistry and Material Technology of Ministry of Education, Hangzhou Normal University, Hangzhou 311121, China

**Keywords:** covalent organic framework, facile synthesis, photocatalyst, dye degradation, nanocomposite

## Abstract

Covalent organic frameworks (COFs), as a novel crystalline porous adsorbent, have been attracting significant attention for their synthesis and application exploration due to the advantages of designability, stability, and functionalization. Herein, through increasing the concentration of the acid catalyst, a facile solution-refluxing synthesis method was developed for the preparation of a three-dimensional dynamic COF material, COF-300, with high yields (>90%) and high space–time yields (>28 kg m^–3^ day^–1^). This synthesis method not only permits gram-scale synthesis, but also yields products that well maintain porosity and unique guest-dependent dynamic behavior. Moreover, the catalytic activity of COF-300 as a metal-free photocatalyst was explored for the first time. Under 365 nm ultra-violet light irradiation, COF-300 can effectively catalyze the dye degradation (>99%) in wastewater with good recyclability. By adding magnetic Fe_3_O_4_ nanoparticles into the solution-refluxing synthesis of COF-300, Fe_3_O_4_/COF-300 nanocomposites can be obtained and used as magnetically recyclable photocatalysts, demonstrating the superiority of this facile synthesis procedure. Our study provides new insights for the preparation of COF materials and a constructive exploration for their water treatment application.

## 1. Introduction

Covalent organic frameworks (COFs) are an emerging class of crystalline covalent organic polymers combining porosity and an ordered structure [1,2,3,4]. With their designable structures and functionalities, COFs have attracted considerable attention in gas storage and separation [5,6], catalysis [7,8], sensing [9,10], conductivity [11,12], and so on. Since the invention of COFs in 2005 [13], the synthesis of COFs with extensive structural diversity has been witnessed. Recently, series of imine-linked COFs with unique dynamic structural transformations (e.g., COF-300 [14], COF-320 [15], LZU-301 [16], COF-117 [17], 3D-CageCOF-1 [18], and FCOF-5 [19]) have gained widespread interest because they are considered as ideal materials for smart stimuli-response. However, the difficulty in the high-quality and scalable preparation of COFs still limits their application promotion [2,3,20]. Although the substantial amount of effort that has been devoted to the synthesis of COFs and some new synthetic methods (e.g., microwave-assisted synthesis [21], ionothermal synthesis [22], mechanochemical synthesis [23], interfacial synthesis [24], and ultrasound-assisted synthesis [25]) has obtained substantial achievements [20], the solvothermal synthesis method is still the first choice to produce high-quality COFs [2]. The conventional solvothermal synthesis methods generally require the degassing of the reaction substrates and solvents via freeze–pump–thaw cycles, followed by sealing in a vessel and heating at high temperature for long reaction time (c.a. 3–7 days) [2,20]. Such harsh reaction conditions restrict the preparation of COFs on a large scale. Previously, we reported the ventilation-vial synthesis method [26], which avoids the complicated operation, but still requires 2–3 days of reaction time and the yield is less than 80%. Therefore, exploring reproducible and scalable COF synthesis methods with high yields and high space–time yields is still highly desired and challenging.

Organic dyes are used in industrial production in enormous quantities (more than 70,000 tons/year) and are the most major water pollutants [27]. By virtue of mild reaction conditions and low-energy consumption, photodegradation technology towards dyes becomes one of the current preferred pathways of wastewater treatment [28], in which photocatalysts play a critical role. Thanks to the high charge carrier mobility guaranteed by large p-conjugated structures and the sufficiently exposed catalytic sites induced by large specific surface areas, COFs have demonstrated great potential for photocatalytic applications and environmental remediation [29,30,31], including water splitting [32,33], CO_2_ reduction [34,35], organic transformation [36,37,38], and pollution degradation [39]. The stability of COFs also contributes to avoiding corrosion of photoactive units and improving recyclability [40]. Compared with traditional metal catalysts, COFs, as metal-free photocatalysts for industrial dye degradation, not only can reduce the consumption of metal resources and eliminate the secondary environmental pollution, but also are more promising for sustainable development [7,36]. In addition, the rapid recovery of photocatalysts is also an important consideration for their utilization in dye photodegradation. The fabrication of COFs into multifunctional composites or devices would be an effective solution [41,42,43,44]. This requires facile synthetic methods for the composites or devices fabrication.

Herein, a dynamic three-dimensional (3D) imine-linked COF, COF-300 [14,26,45,46], was selected as the target. A facile solution-refluxing synthesis method was employed to prepare COF-300. This synthetic method is not only high in yield and space–time yield, but also convenient to operate and scalable. The gas sorption behaviors and solvent-dependent dynamic behaviors confirmed the credible quality of the products. The remarkable dye photodegradation performance of COF-300 was also investigated. It is worth mentioning that the magnetic COF-300 composites with no loss of dye photodegradation performance and facilitate recovery can be easily constructed by this synthetic method.

## 2. Results and Discussion

### 2.1. Facile Solution-Refluxing Synthesis of COF-300

**Figure 1:**
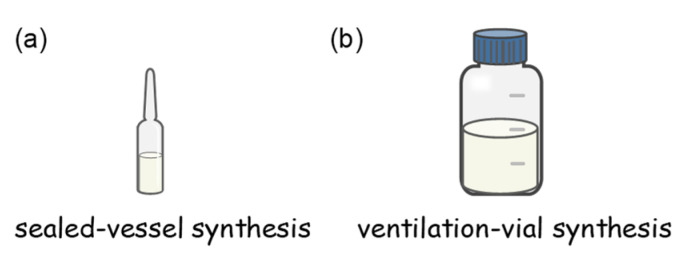
Schematic illustration of (**a**) sealed-vessel and (**b**) ventilation-vial synthesis methods.

COF-300, formed by the dehydration of 4-(tris(4-aminophenyl)methyl)benzenamine (TAM) and terephthalaldehyde (TPA), is the first crystalline porous 3D framework material constructed from imine linkages [14]. Subsequently, a large number of imine-linked COFs have been reported and widely used [2]. Most of these imine-linked COFs were synthesized by the sealed-vessel synthesis method under inert conditions initially (Figure 1a). Recently, we reported the ventilation-vial synthesis method for the synthesis of COF-300 (Figure 1b) [26]. Through adding a suitable ratio of poor solvents for optimizing the crystallization conditions, COF-300 can be successfully synthesized without degassing and sealed-vessel operation.

**Figure 2:**
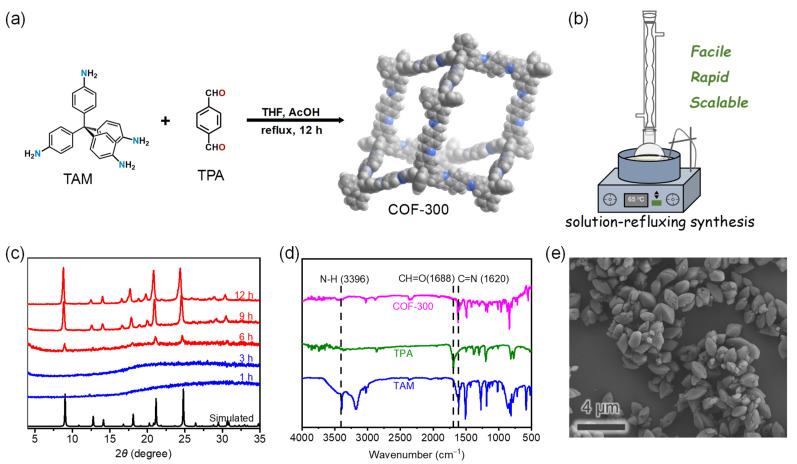
(**a**) Synthetic route of COF-300; (**b**) Schematic illustration of solution-refluxing synthesis method; (**c**) Reaction time-dependent PXRD patterns of products in solution-refluxing synthesis method; (**d**) The IR spectra of TAM, TPA and solution-refluxing synthesized COF-300; (**e**) The SEM photograph of solution-refluxing synthesized COF-300.

Using the sealed-vessel and ventilation-vial synthesis methods, the crystallinity of the COF-300 products for different reaction times were monitored with powder X-ray diffraction (PXRD), and then the yields of the crystalline products were determined (Table 1 and Figures S1 and S2). For the sealed-vessel synthesis method, although the formation of products with good crystallinity was observed after a reaction time of 12 h, the yield was low (<25%) and the atomic economy was unsatisfactory. The yield for 48 h reaction time of increased to 66%, and no significant yield increase was observed when the reaction time was extended to 72 h. For the ventilation-vial synthesis method, products with good crystallinity can be obtained with a reaction time of 48–72 h, but the yield still did not exceed 80%. The space–time yields of both synthesis methods did not exceed 10 kg m^−3^ day^−1^. In order to achieve a reproducible and easy-to-operate synthesis, we consider the reaction in a flask under open-air condition with reference to the general organic synthesis operation (Figure 2). The reaction substrates can be mixed well by stirring and refluxing the solution. Through increasing the catalyst concentration of the aqueous acetic acid solution to 12 mol//L, the formation of crystalline products was observed with a reaction time of 6 h (Figure 2c). The yield for a 9 h reaction time reached 90%, corresponding to a space–time yield of 37.7 kg m^−3^ day^−1^. The experimental PXRD patterns of the products with 9–12 h reaction time matched well with the simulated one (Figure 2c) [26]. The infrared (IR) spectra showed that the product was almost free of unreacted amino and aldehyde groups (Figure 2d). Scanning electron microscopy (SEM) photograph revealed that the product morphology was regular and rice-like, consistent with previous reports (Figure 2e) [26]. The thermogravimetric analysis (TGA) curve showed no significant weight loss of the vacuum-dried sample below 400 °C (Figure S3), suggesting the good thermal stability of COF-300 we prepared, which was further confirmed by variable-temperature PXRD measurement (Figure S4). These characterization results indicated that COF-300 can be successfully synthesized with high yields and high space–time yields by the solution-refluxing synthesis method (Table S1).

### 2.2. Gram-Scale Synthesis and Dynamic Behaviors of COF-300

**Figure 3:**
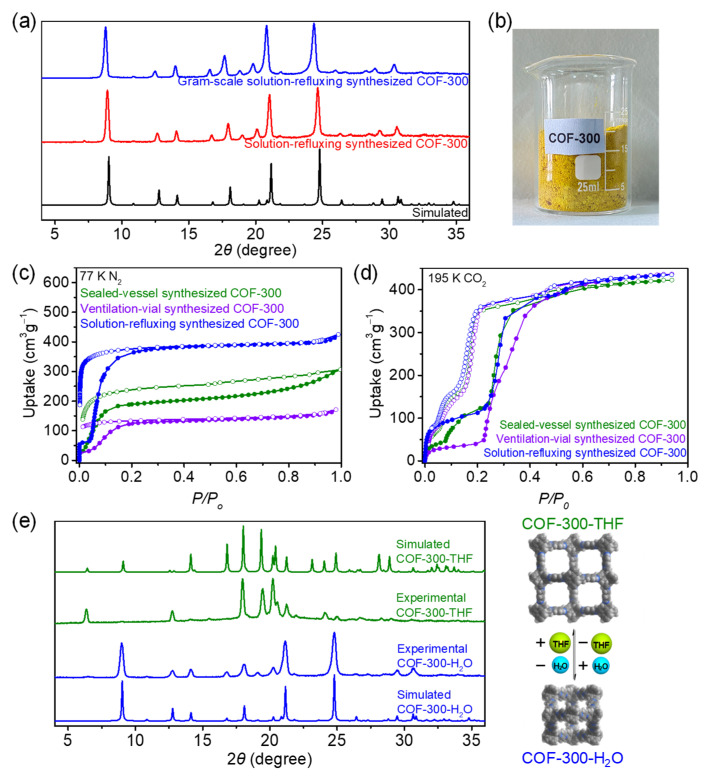
(**a**) PXRD patterns and (**b**) appearance of gram-scale solution-refluxing synthesized COF-300; (**c**) Nitrogen sorption isotherms at 77 K and (**d**) carbon dioxide sorption isotherms at 195 K; (**e**) The solvent-dependent PXRD patterns of COF-300.

To explore the reproducibility and scalability of the solution-refluxing synthesis method, we scaled up the reaction by 10 times (Figure S5). Satisfactorily, the yield of the gram-scale synthesis exceeded 90% without decrease and more than 2 g of product can be obtained in a single synthesis procedure (Figure S6). PXRD showed that the product had good crystallinity and purity (Figure 3a). The appearance of the product was very homogeneous in color (Figure 3b). Such excellent reproducibility and scalability should be attributed to the fact that the synthetic system of the solution-refluxing synthesis method is much more homogeneous than the stationary solvothermal reaction.

The permanent porosity and guest-dependent dynamic behaviors are the most remarkable features of COF-300. Nitrogen sorption isotherms at 77 K (Figure 3c) and carbon dioxide sorption isotherms at 195 K (Figure 3d) were tested for the products of all three synthetic methods (sealed-vessel, ventilation-vial, and solution-refluxing synthesis). All sorption isotherms exhibited sorption hysteresis behavior but were not identical. At 77 K, the nitrogen saturation uptake (*P*/*P*_0_ = 0.9) of COF-300 was 265 cm^3^/g for sealed-vessel synthesis, 155 cm^3^/g for ventilation-vial synthesis, and 400 cm^3^/g for solution-refluxing synthesis. The COF-300 product of solution-refluxing synthesis seems to be easier to expand during gas sorption. At 195 K, the carbon dioxide saturation uptakes (*P*/*P*_0_ = 0.9) of COF-300 obtained all three synthetic methods were similar (c.a. 430 cm^3^/g) and corresponded to a pore volume of 0.75 cm^3^/g, which matched well with that of the solvated structure of COF-300 [26]. Although all samples showed adsorption jumping behavior at *P*/*P*_0_ = 0.22, the uptake of ventilation-vial synthesized COF-300 was only 48 cm^3^/g before the jumping while that of solution-refluxing synthesized COF-300 reached 122 cm^3^/g. Intermediately, sealed-vessel synthesized COF-300 had an extra step adsorption jumping at *P*/*P*_0_ = 0.08. Before the jumping, its uptake was close to that of ventilation-vial synthesized COF-300. After the jumping, its uptake caught up with that of solution-refluxing synthesized COF-300. These gas sorption isotherms in combination showed that the expansion tendency of COF-300 obtained by the three synthetic methods follows the order: ventilation-vial < sealed-vessel < solution-refluxing. These phenomena suggested that the dynamic behavior of COF-300 can be modulated by the synthetic method [47,48].

Besides the gases, the dynamic behavior of COF-300 can be triggered by solvents. PXRD showed that when the activated COF-300 sample was placed in a tetrahydrofuran (THF) vapor atmosphere, COF-300 would expand (Figure 3e). When the expanded COF-300 containing THF was placed in a water vapor atmosphere, COF-300 underwent contraction. This reversible dynamic structural transition behavior also reflected the high quality of solution-refluxing synthesized COF-300.

### 2.3. Photocatalytic Dye Degradation Performance of COF-300

**Figure 4:**
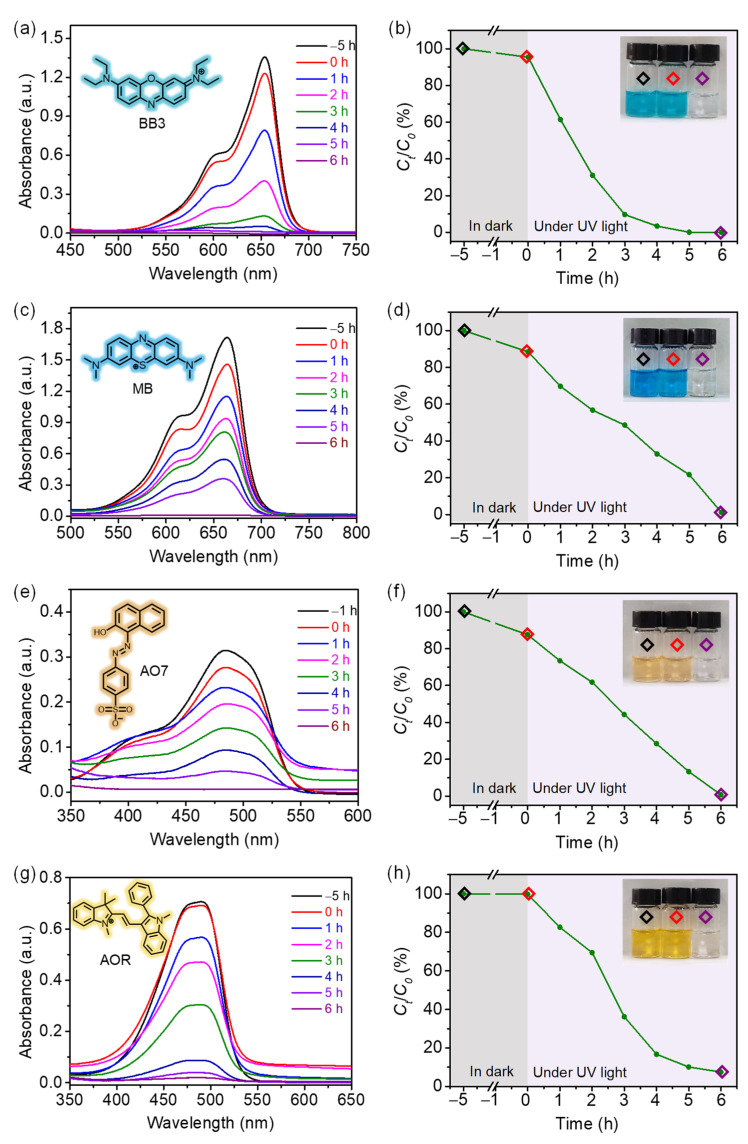
Reaction time-dependent UV–vis absorption spectra and concentration variations of (**a**,**b**) BB3, (**c**,**d**) MB, (**e**,**f**) AO7, and (**g**,**h**) AOR with the presence of COF-300 (Inset shows the photographs of the dye solutions. Black: blank; red: 5 h in dark; purple: 6 h under UV light irradiation).

**Figure 5:**
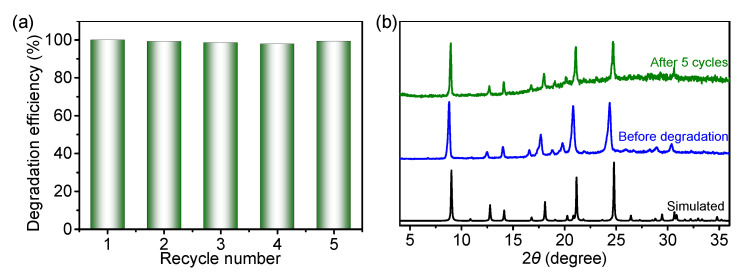
(**a**) Recyclability of COF-300 for BB3 photocatalytic degradation; (**b**) PXRD patterns of COF-300 after cyclic degradation experiments.

Inspired by the recent progress of COFs in metal-free photocatalysis application [29,30,31], the photodegradation dye performance of COF-300 was tested. Four dyes with different charges and chromogenic groups were selected as photodegradation targets, including Basic Blue 3 (BB3), Methylene Blue (MB), Acid Orange 7 (AO7), and Astrazon Orange R (AOR). A series of dye aqueous solutions with different concentrations were prepared for the ultraviolet-visible (UV–vis) absorption spectroscopy standard curves (Figures S7–S10). A small amount (0.1 wt%) of COF-300 was placed into the dye solution (8 ppm) and the concentration change of the dye solution was monitored by UV–vis absorption spectroscopy (Figure 4). While there was no significant fading of the dye solution in dark with COF-300 for 5 h or under UV light irradiation without the catalyst for 6 h (Figure S11) [49], there was an almost complete discoloration under UV light irradiation with COF-300 for 6 h. The dye degradation efficiencies were determined using UV–vis absorption spectroscopy, in which >99% for BB3, >98% for MB, >99% for AO7, and >92% for AOR (Table S2). The reusability of this efficient dye photodegradation performance was confirmed by five recycle degradation experiments (Figure 5a). PXRD data also displayed that COF-300 maintained its crystallinity after photocatalytic dye degradation (Figure 5b).

### 2.4. Photocatalytic Dye Degradation Mechanism of COF-300

**Figure 6:**
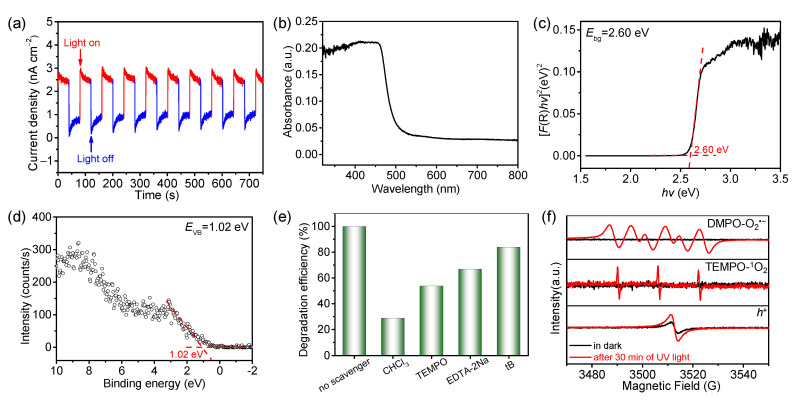
(**a**) Photocurrent responses, (**b**) UV–vis diffuse reflectance spectrum, (**c**) Tauc plot analysis, and (**d**) VB-XPS spectrum of COF-300; (**e**) Effect of scavengers on BB3 degradation efficiencies (TEMPO: 2,2,6,6-tetramethyi-1-piperidinyloxy; EDTA-2Na: ethylenediaminetetraacetic acid disodium salt; tB: tert-butanol); (**f**) ESR spectra for the detection of O_2_**^•^**^–^, ^1^O_2_, and *h*^+^.

The transient photocurrent responses of COF-300 in on–off cycles under UV–vis light (*λ* = 300–1100 nm) irradiation showed its photogenerated electrons–holes ability (Figure 6a). According to the UV–vis diffuse reflectance spectrum (Figure 6b), the band gap of COF-300 was estimated to be 2.60 eV by utilizing Tauc plot analysis (Figure 6c). The valence band (VB) edge of COF-300 was located at 1.02 eV evaluated from its VB X-ray photoelectron spectroscopy (VB-XPS) signal (Figure 6d). To elucidate the photocatalytic dye degradation mechanism of COF-300, the radical trapping experiments were performed to determine the possible active species by adding radical scavengers in the photocatalysis system (Figure 6e). The degradation efficiencies of BB3 decreased to 28.8%, 53.7%, and 66.9% after adding CHCl_3_ (a superoxide radical O_2_**^•^**^–^ scavenger), TEMPO (a singlet Oxygen ^1^O_2_ scavenger), and EDTA-2Na (a hole *h*^+^ scavenger), respectively, while just a slight decrease was observed after adding tB (a hydroxyl radical HO**^•^** scavenger). Electron spin resonance spectroscopy (ESR) experiments were conducted to identify the radicals generated with the presence of COF-300 (Figure 6f). Consistently, the signals of O_2_**^•^**^–^, ^1^O_2_, and *h*^+^ were detected in ESR spectra of COF-300 after 30 min UV light irradiation.

**Figure 7:**
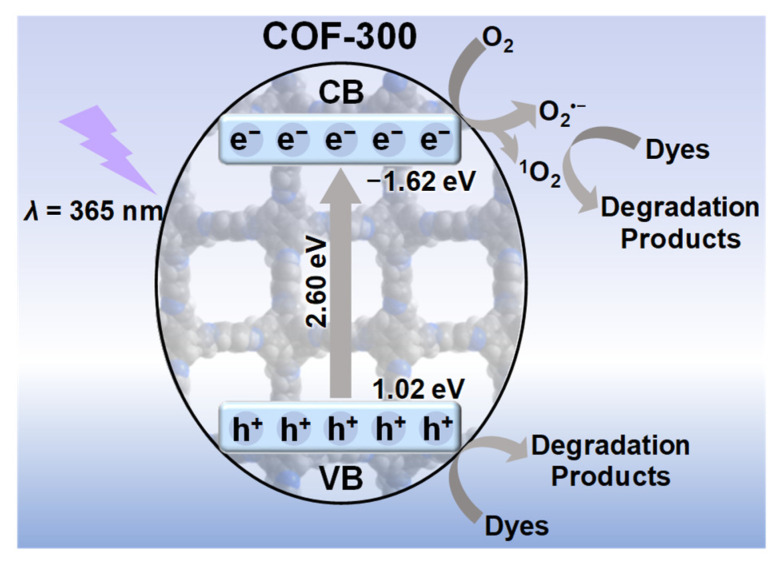
The schematic illustration for the generation of reactive species and photocatalytic dye degradation mechanism of COF-300.

The above results clearly indicated that O_2_**^•^**^–^, ^1^O_2_, and *h*^+^ were the main active species for the photocatalytic dye degradation of COF-300. A probable mechanism for the photocatalytic dye degradation was proposed (Figure 7). The electron/hole pairs of COF-300 were generated and separated by UV light excitation, assisting the conversion of Oxygen to singlet Oxygen. While ^1^O_2_ and *h*^+^ directly promoted the dye degradation, *e*^–^ transfer to oxygen forming O_2_**^•^**^–^ involved in the degradation of dyes. COF-300 is expected to be used to improve the selectivity of mild oxidation reactions due to no generation of the highly oxidizing reactive species HO**^•^** (*E*[HO**^•^**/H_2_O] = 2.33 V).

### 2.5. COF-300 Composite for Photocatalytic Dye Degradation

**Figure 8:**
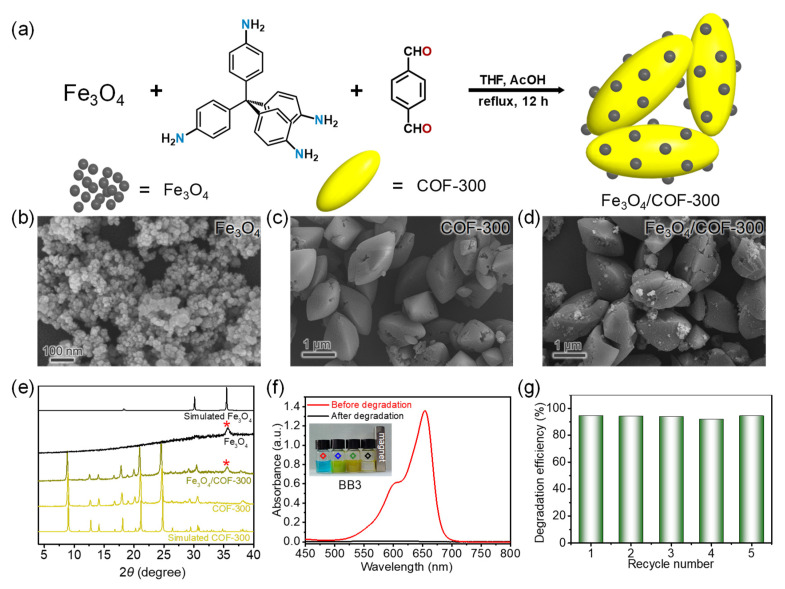
(**a**) Synthetic route of Fe_3_O_4_/COF-300 nanocomposites; (**b**–**d**) SEM images of Fe_3_O_4_, COF-300 without adding Fe_3_O_4_, and Fe_3_O_4_/COF-300; (**e**) PXRD pattern comparison for Fe_3_O_4_/COF-300; (**f**) UV–vis absorption spectra of BB3 aqueous solutions (Inset shows the photographs of the BB3 solutions. Red: blank; blue: adding Fe_3_O_4_/COF-300 nanocomposites; green: after 6 h UV light irradiation; black: after magnetic recovery); (**g**) Recyclability of Fe_3_O_4_/COF-300 for BB3 photocatalytic degradation.

Constructing photocatalyst composites as well as devices for the ready recovery after the environmental remediation process is important for the promotion of photocatalyst applications [41,42,43,44]. The homogeneity of the solution-refluxing reaction system facilitates the preparation of composites. We tried to prepare Fe_3_O_4_/COF-300 composites by adding Fe_3_O_4_ nanoparticles to the solution-refluxing synthesis process of COF-300 (Figure 8a). Without changing other synthesis procedures, magnetic COF-300-based composites were successfully obtained (Figure S12). SEM images showed that Fe_3_O_4_ nanoparticles we prepared were uniform in morphology with size concentration of ~10 nm (Figure 8b). The morphology and size (~1 mm) of COF-300 were not affected by the addition of Fe_3_O_4_ nanoparticles (Figure 8c,d). Owing to refluxing and stirring during the synthesis process and the interface interaction between Fe_3_O_4_ and COF-300, Fe_3_O_4_ nanoparticles were dispersed and embedded on the surface of COF-300 to form Fe_3_O_4_/COF-300 composites. PXRD pattern comparison confirmed the presence as well as the crystallinity of COF-300 and Fe_3_O_4_ in Fe_3_O_4_/COF-300 composites (Figure 8e). Elemental analyses and inductively coupled plasma atomic emission spectrometry identified the content of COF-300 in Fe_3_O_4_/COF-300 composites up to 91% (Tables S3 and S4), which is favorable for the retention of photocatalytic performance. Although the dye photodegradation performance of Fe_3_O_4_ is poor (Figure S11) [50], it can provide a magnetic recovery pathway for photocatalysts. As expected, the UV–vis absorption spectroscopy monitoring results (Figure 8f,g) also showed that Fe_3_O_4_/COF-300 composites, not only inherited the catalytic performance and recyclability of COF-300 well, but also benefited from the magnetic properties conferred by Fe_3_O_4_ making it easy to be recovered.

## 3. Materials and Methods

### 3.1. Materials and Measurements

All reagents were commercially available and used without purification, except otherwise specified. PXRD data were collected using a Rigaku MiniFlex 600 diffractometer (Rigaku, Tokyo, Japan) (Cu K*α*, *λ* = 1.5418 Å). Variable-temperature PXRD data were collected in a Rigaku Ultima IV diffractometer (Rigaku, Tokyo, Japan) (Cu K*α*, *λ* = 1.5418 Å) under N_2_ flow. TGA curves were performed on a TA Q50 apparatus (TA, USA) under nitrogen flow. SEM images were obtained from a Zeiss Gemini 300 field emission electron microscopy (Zeiss, Jena, Germany). Inductively coupled plasma atomic emission spectra were measured with a Shimadzu ICPE-9000 full-spectrum ICP-emission spectrometer (Shimadzu, Kyoto, Japan). Elemental analyses were measured using an Elementar Vario EL cube elemental analyzer (Elementar, Langenselbold, Germany). Gas sorption isotherms were collected on a BSD-PM2 or BSD-PMC sorption analyzer (BSD, Beijing, China). The photocurrent densities were measured under light irradiation of quartz optical fiber (*λ* = 300–1100 nm) in a Perfectlight PEC 1000 photoelectric catalytic testing system (Perfectlight, Beijing, China) and a Chenhua CHI 660E electrochemical workstation (Chenhua, Shanghai, China). ESR measurements were performed on a BRUKE EMXPLUS electron paramagnetic resonance spectrometer (BRUKE, Rheinstetten, Germany) in dark or after 30 min UV light irradiation. VB-XPS was collected on an Escalab 250XiXPS energy spectrometer using Al Kα radiation (ThermoFisher, Waltham, MA, USA). Solid-state UV–vis diffuse reflectance spectrum was measured using a Lambda 950 UV–vis spectrometer from 320 to 800 nm (PerkinElmer, Waltham, MA, USA). The absorbances of dye solutions were measured by using a SHIMADZU UV-1780 UV–vis spectrophotometer (Shimadzu, Kyoto, Japan). IR spectra were recorded with a Thermo IR200 Fourier transform IR spectrometer (Thermo Nicolet, Waltham, MA, USA).

### 3.2. Sealed-Vessel Synthesis of COF-300

The sealed-vessel synthesis was performed according to the literature [14]. A mixture of TPA (24 mg, 0.178 mmol) and TAM (40 mg, 0.104 mmol) was dissolved in anhydrous 1,4-dioxane (2 mL) within a glass tube under sonication. Then aqueous acetic acid (0.2 mL, 3 mol/L) was added into the mixture, which was degassed via freeze–pump–thaw cycles. After the glass tube was sealed with a torch, the mixture was placed in an oven at 120 °C for 48 h. The products were collected by filtration, washed with fresh THF, and then dried under vacuum at 120 °C for 12 h to obtain yellow powder of COF-300 (34 mg, 66% yield based on TPA).

### 3.3. Ventilation-Vial Synthesis of COF-300

The ventilation-vial synthesis was performed according to the literature [26]. A mixture of TPA (12 mg, 0.089 mmol) and TAM (20 mg, 0.052 mmol) was dissolved in anhydrous 1,4-dioxane (1.6 mL) and cyclohexane (0.4 mL) within a 4 mL vial under sonication. Then aqueous acetic acid (0.4 mL, 6 mol/L) was added into the vial, which was capped and placed in an oven at 65 °C for 48 h. The products were collected by filtration, washed with fresh THF, and then dried under vacuum at 120 °C for 12 h to obtain yellow powder of COF-300 (19 mg, 74% yield based on TPA).

### 3.4. Solution-Refluxing Synthesis of COF-300

A mixture of TPA (173 mg, 0.455 mmol) and TAM (104 mg, 0.776 mmol) was dissolved in THF (10 mL) within a 50 mL flask at 65 °C. Then, aqueous acetic acid (4 mL, 12 mol/L) was added, and the solution was kept refluxing and stirred vigorously for 12 h. The products were collected by filtration, washed with fresh THF, and then dried under vacuum at 120 °C for 12 h to obtain yellow powder of COF-300 (202 mg, 92% yield based on TPA).

### 3.5. Gram-Scale Solution-Refluxing Synthesis of COF-300

Scale up the above solution-refluxing synthesis by 10 times. A mixture of TPA (1.73 g, 4.55 mmol) and TAM (1.04 g, 7.76 mmol) was dissolved in THF (100 mL) within a 250 mL flask. Then aqueous acetic acid (40 mL, 12 mol/L) was added. The other procedure was performed as above to obtain yellow powder of COF-300 (2.03 mg, 93% yield based on TPA).

### 3.6. Synthesis of Fe_3_O_4_ Nanoparticles

Fe_3_O_4_ nanoparticles was synthesized according to the literature [51]. A mixture of FeCl_2_ (0.5 g, 3.94 mmol) and FeCl_3_ (20 mg, 0.052 mmol) was dissolved in deionized water (20 mL). The solution was heated and stirred for 10 min at 40 °C. Then, concentrated ammonia (~3 mL, 25%~28%) was added drop-by-drop to adjust the pH to 8.0~9.0 and heat the solution at 80 °C for 1 h. After washing with water and ethanol for 3 times, the solid products were separated with a magnet and dried in an oven at 70 °C. The obtained black powder was Fe_3_O_4_ nanoparticles (390 mg, 88% yield).

### 3.7. Solution-Refluxing Synthesis of Fe_3_O_4_/COF-300

Refer to the above synthesis procedure of 3.4. After the addition of acetic acid, Fe_3_O_4_ nanoparticles (50 mg) were added. The other procedure was performed as above to obtain yellow powder of Fe_3_O_4_/COF-300 (220 mg, 90% yield based on TPA).

### 3.8. Photocatalytic Dye Degradation

COF-300 or Fe_3_O_4_/COF-300 samples (10 mg) were placed in the dye solutions (8 ppm, 10 mL), and the UV−vis spectra of the dye solutions were tested after different reaction time in dark or under UV light (*λ* = 365 nm, 300 W).

## 4. Conclusions

In summary, we developed a solution-refluxing synthesis method for the preparation of a dynamic COF material, COF-300. The synthetic method is scalable and can achieve yields of more than 90% and space–time yields of more than 28 kg m^−3^ day^−1^. The product obtained by this method can expand towards gas at lower pressure than that obtained by previous synthetic methods. Thanks to the homogeneity of the reaction system, it is convenient for the preparation of COF-300 composites using the solution-refluxing synthesis method. The photocatalytic properties of COF-300 were investigated for the first time. Fe_3_O_4_/COF-300 composites exhibited satisfactory results in dye wastewater treatment. Our study provided a positive exploration for the preparation of COF materials and established the foundation for the promotion of COF applications.

## Figures and Tables

**Table 1:** Time-dependent syntheses of COF-300 via different synthetic method.

Synthetic Method	Time (h)	Crystallinity	Yield(%)	Space–Time Yield (kg m^−3^ day^−1^)
Sealed-vessel Synthesis	1	amorphous	\	\
	3	amorphous	\	\
	6	moderate	20	16.7
	12	good	23	10.0
	24	good	35	7.5
	48	good	66	7.1
	72	good	69	5.0
Ventilation-vial Synthesis	1	amorphous	\	\
	3	amorphous	\	\
	6	amorphous	\	\
	12	moderate	62	13.3
	24	moderate	74	7.9
	48	good	74	3.9
	72	good	78	2.8
Solution-refluxing Synthesis	1	amorphous	\	\
	3	amorphous	\	\
	6	moderate	75	47.7
	9	good	90	37.7
	12	good	92	28.9

## Data Availability

All the raw data of this research can be obtained from the corresponding authors upon reasonable request.

## Supplementary Materials

The following supporting information can be downloaded at: https://www.mdpi.com/article/10.3390/molecules27228002/s1, Figures S1–S12: PXRD, TG, UV–vis absorption spectra, and photographs; Tables S1–S4: Yields, space-time yields and dye photodegradation performance comparison, elemental analyses and inductively coupled plasma atomic emission spectrometry analyses [15,19,52,53,54,55,56,57,58,59,60,61,62,63,64,65,66,67,68].

## Sample Availability

Samples of the compounds are not available from the authors.
