# Supplementary material for: Facile Solution-Refluxing Synthesis and Photocatalytic Dye Degradation of a Dynamic Covalent Organic Framework
Source: Molecules. 2022 Nov 18;27(22):8002. doi: 10.3390/molecules27228002 (PMC9697432; doi:10.3390/molecules27228002)
Supplement: Supplementary file 1 [file molecules-27-08002-s001.zip › molecules-2036290-supplementary.pdf]

Supplementary Materials

Facile Solution-Refluxing Synthesis and Photocatalytic Dye Degradation of a Dynamic Covalent Organic Framework

Xiaolian Wang 1, Yiying Sun 1, Yonghong Xiao 1, Xiaoxian Chen 2,*, Xiaochun Huang 1,2 and Haolong Zhou 1,3,*

1 Department of Chemistry and Key Laboratory for Preparation and Application of Ordered Structural Materials of Guangdong Province, Shantou University, Shantou 515063, China

2 Chemistry and Chemical Engineering Guangdong Laboratory, Shantou 515031, China

3 Key Laboratory of Organosilicon Chemistry and Material Technology of Ministry of Education, Hangzhou Normal University, Hangzhou 311121, China

* Correspondence: chenxx@ccelab.com.cn (X.C.); hlzhou@stu.edu.cn (H.Z.)

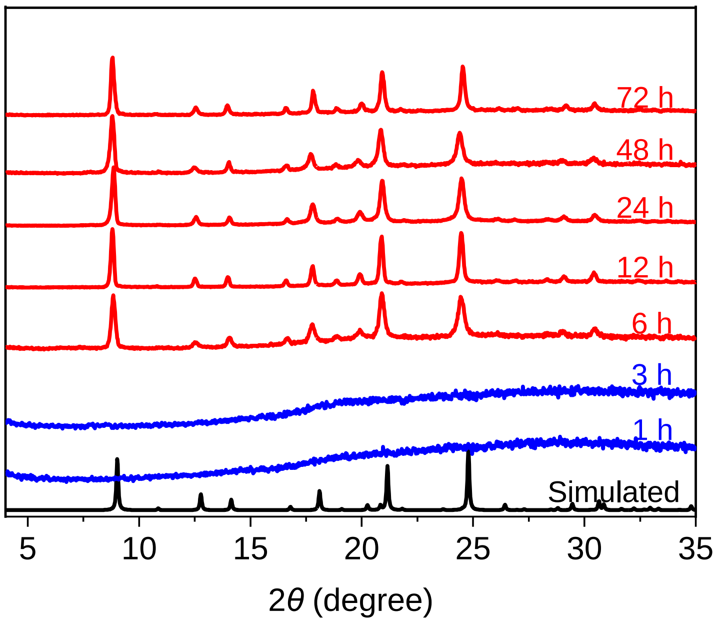

Figure S1. Reaction time-dependent PXRD patterns of products in sealed-vessel synthesis method.

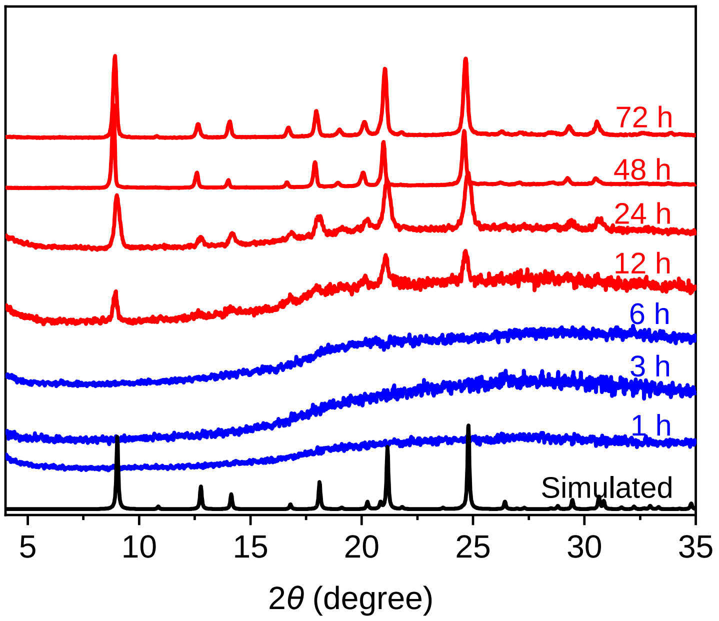

Figure S2. Reaction time-dependent PXRD patterns of products in ventilation-vial synthesis method.

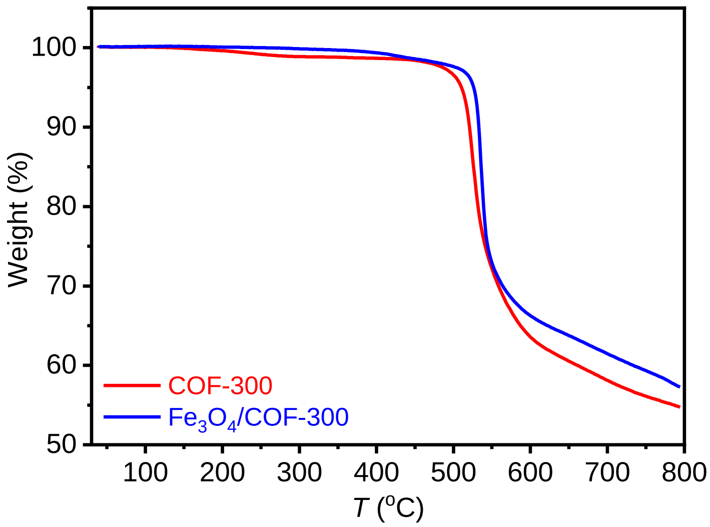

Figure S3. TGA curves of COF-300 and Fe_3O_4/COF-300.

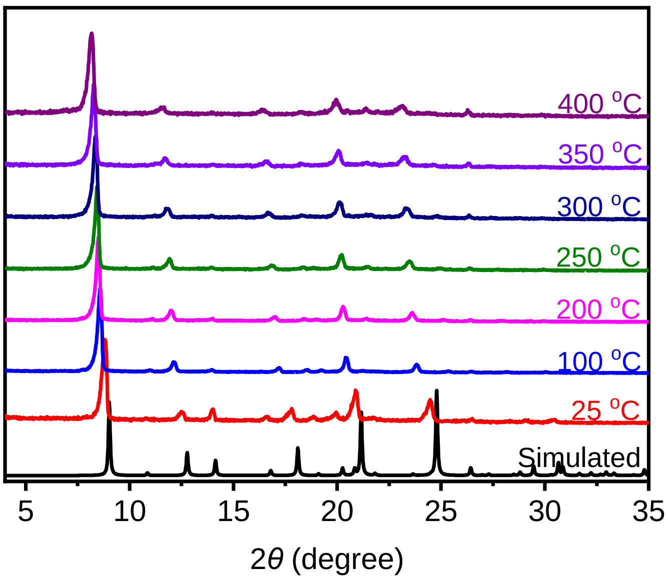

Figure S4. Variable-temperature PXRD patterns of COF-300.

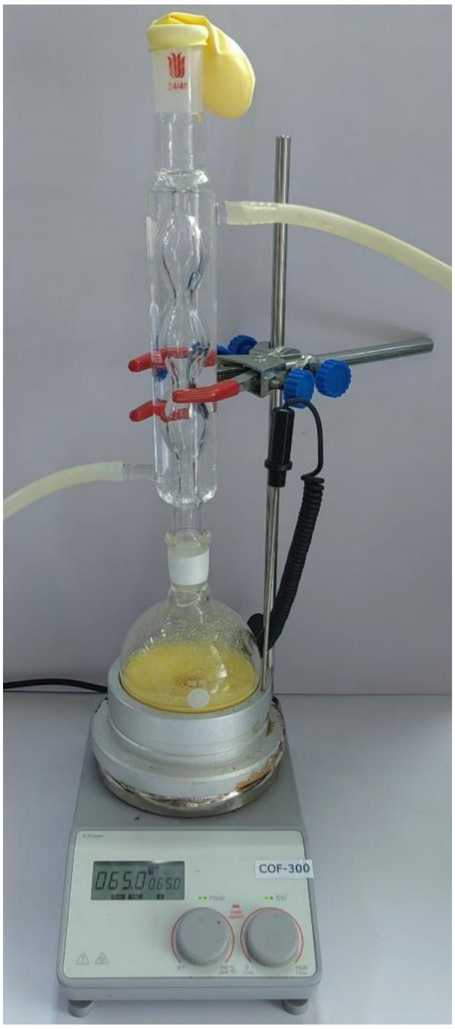

Figure S5. Laboratory setup for gram-scale solution-refluxing synthesis of COF-300.

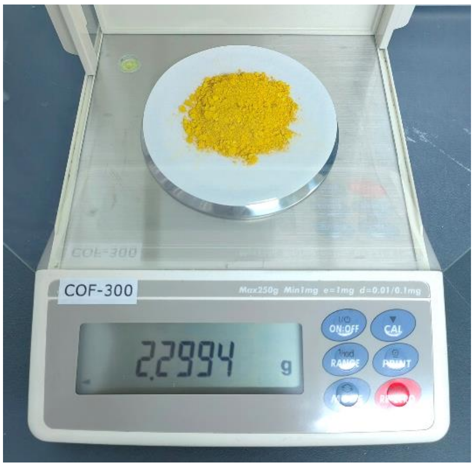

Figure S6. Products obtained from a single gram-scale solution-refluxing synthesis procedure of COF-300.

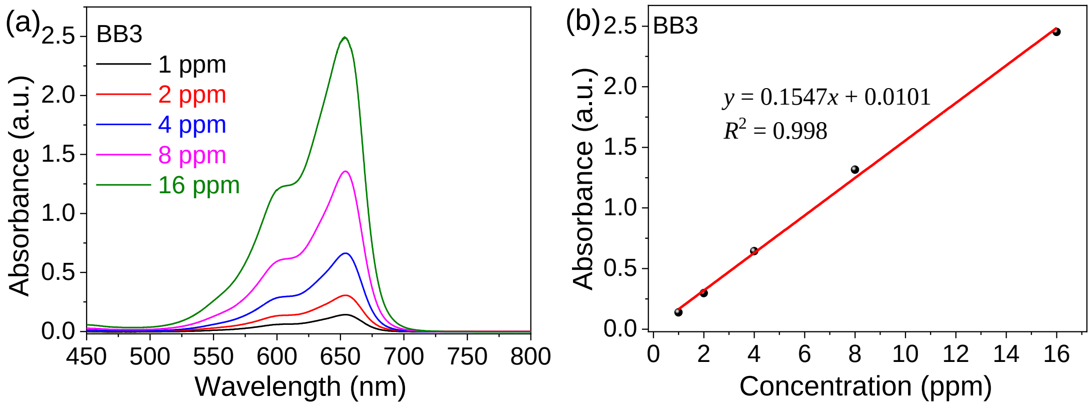

Figure S7. The UV-vis absorption spectroscopy standard curve of BB3.

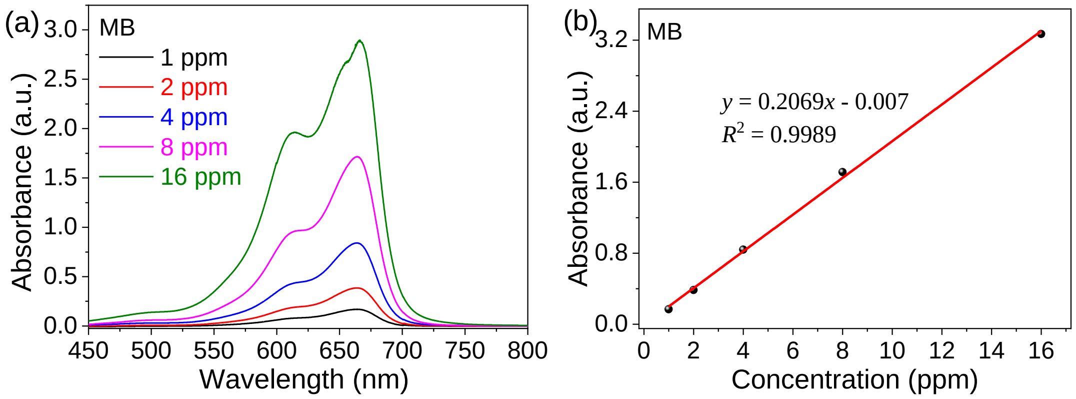

Figure S8. The UV-vis absorption spectroscopy standard curve of MB.

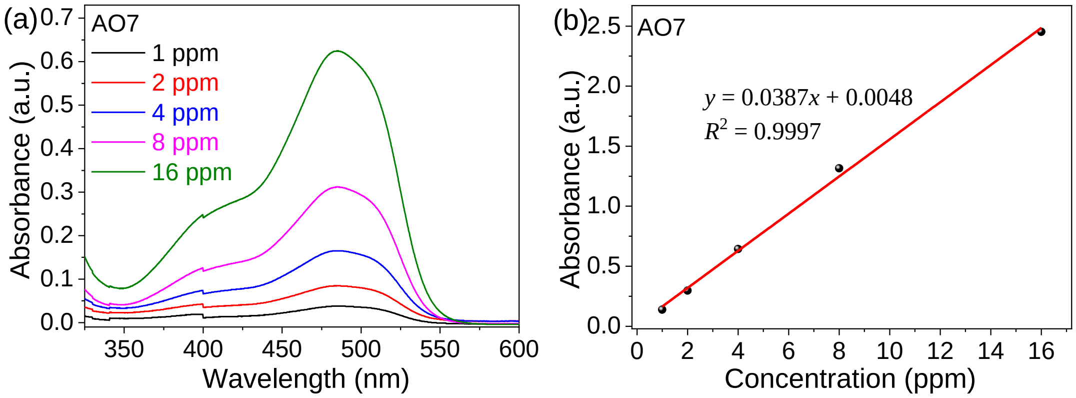

Figure S9. The UV-vis absorption spectroscopy standard curve of AO7.

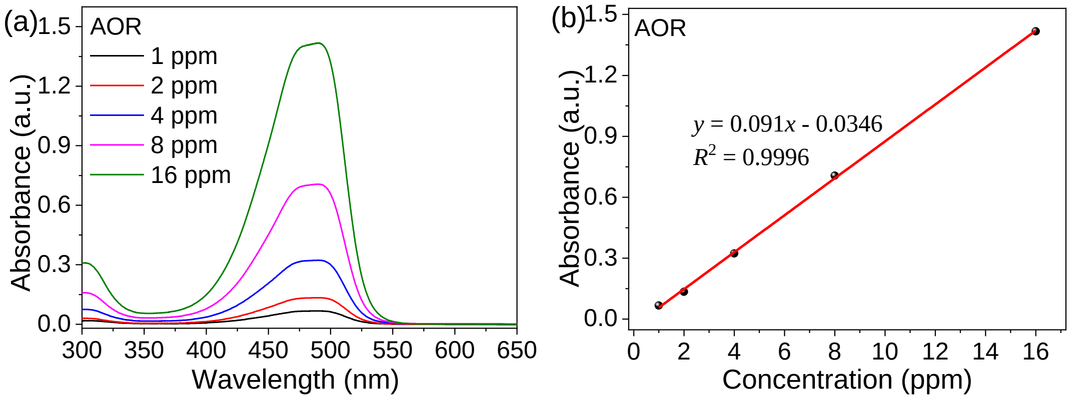

Figure S10. The UV-vis absorption spectroscopy standard curve of AOR.

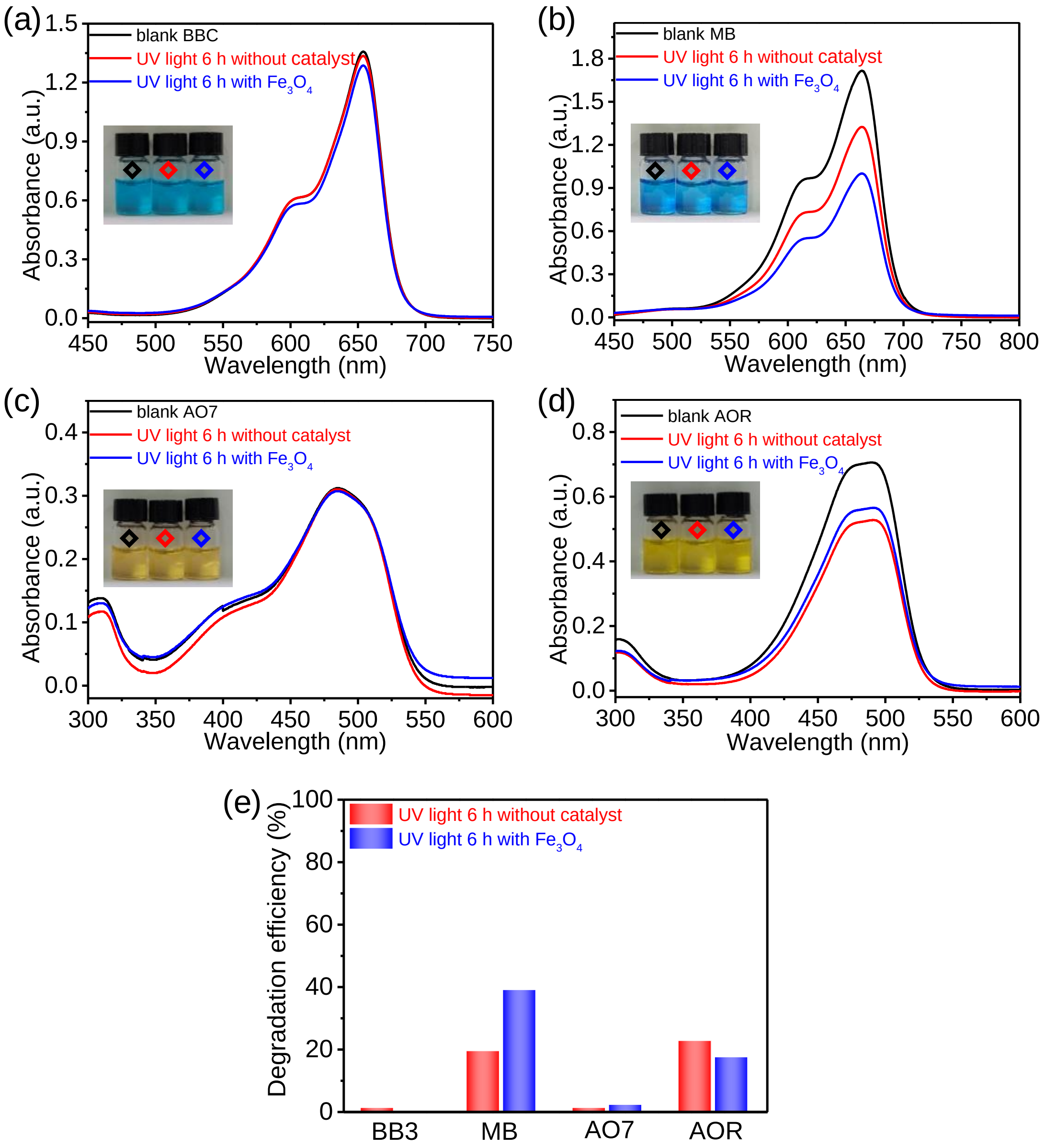

Figure S11. UV-vis absorption spectra of (**a**) BB3, (**b**) MB, (**c**) AO7, and (**d**) AOR under UV light irradiation for 6 hours without catalysts or with the presence of Fe_3O_4 (Inset shows the photographs of the dye solutions. Black: blank; red: without catalyst; blue: with Fe_3O_4); (**e**) Degradation efficiencies of dyes under UV light irradiation for 6 hours without catalysts or with the presence of Fe_3O_4.

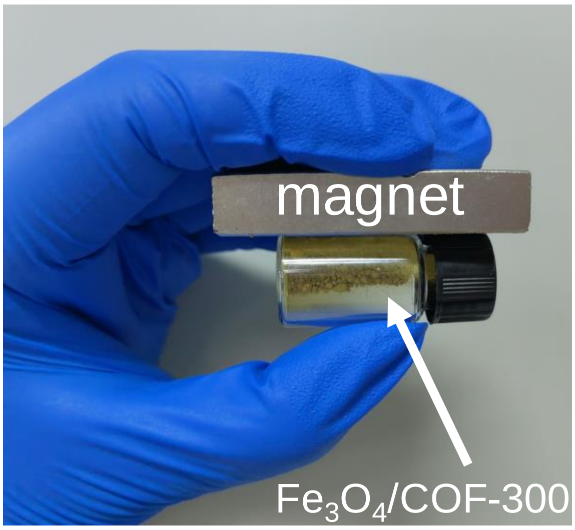

Figure S12. A photograph of Fe_3O_4/COF-300 magnetized by the magnet.

Table S1. The yields and space-time yields of selected imine-linked COFs.

COFs	Temperature (°C)	Yield (%)	Space-time Yield (kg m^{-3} day^{-1})	Ref.
COF-LZU1	120	90	6.7	[1]
COF-366	120	79	4.2	[2]
COF-TpPa-1	120	80	8.5	[3]
COF-320	120	70	6.7	[4]
ETTA-COF	120	90	9.2	[5]
TPB-DMTP COF	120	81	11.6	[6]
3D-OH-COF	120	81	9.7	[7]
3D-TPB-COF-H	100	77	3.1	[8]
FCOF-5	110	75	1.9	[9]
3D-TPB-COF-OMe	120	59	1.3	[10]
COF-300	65	92	28.9	This Work

Table S2. Dye photodegradation performance of selected materials.

Materials	Catalysts	Dyes	Light	Degradation efficiency	Ref.
Inorganic	ZnO	Methyl Violet	UV	60%	[11]
	TiO_2	Methylene Orange	UV	100%	[12]
	$PbWO_4$	Acid Orange II	UV	100%	[13]
Organic	MIL-53+H_2O_2	Methylene Blue	Vis	31%	[14]
	COP/NT+H_2O_2	Methylene Blue	Vis	57%	[15]
Doped	Ce/TiO_2	Methylene Blue	Vis	55%	[16]
	5Fe-TiO_2@TpTa	Methylene Blue	Vis	95%	[17]
	g-C_3N_4/Ag_3VO_4	Basic Fuchsin	Vis	95%	[18]
	Cu_2O/RGO	Methylene Orange	Vis	71%	[19]
This work	COF-300	Basic Blue 3	UV	99%	/

Table S3. Elemental analyses of COF-300 and Fe_3O_4/COF-300.

	Sample	N%	C%	H%	sum%	C/N
Experimental	COF-300	8.71	77.24	5.03	90.98	8.71
	Fe_3O_4/COF-300	7.91	70.31	4.84	83.06	8.88
Theoretical	COF-300·3.5H_2O	8.76	76.99	5.47	91.22	8.78
The content of COF-300 in Fe_3O_4/COF-300		83.06 ÷ 90.98 = 91.29%				

Table S4. Inductively coupled plasma atomic emission spectrometry analyses of COF-300 and Fe_3O_4/COF-300.

Sample	Concentration	Fe concentration	Fe_3O_4 Ratio
$Fe_3O_4 \cdot xH_2O$	10 mg/L	6.34 mg/L	87.4%
Fe_3O_4/COF-300	40 mg/L	2.21 mg/L	7.60%
The content of COF-300 in Fe_3O_4/COF-300		1 - 7.60% ÷ 87.4% = 91.30%	

References

1. Ding, S.Y.; Gao, J.; Wang, Q.; Zhang, Y.; Song, W.G.; Su, C.Y.; Wang, W., Construction of Covalent Organic Framework for Catalysis: Pd/COF-LZU1 in Suzuki-Miyaura Coupling Reaction. *J. Am. Chem. Soc.* **2011**, 133, 19816-19822.
2. Wan, S.; Gandara, F.; Asano, A.; Furukawa, H.; Saeki, A.; Dey, S.K.; Liao, L.; Ambrogio, M.W.; Botros, Y.Y.; Duan, X.F.; Seki, S.; Stoddart, J.F.; Yaghi, O.M., Covalent Organic Frameworks with High Charge Carrier Mobility. *Chem. Mater.* **2011**, 23, 4094-4097.
3. Kandambeth, S.; Mallick, A.; Lukose, B.; Mane, M.V.; Heine, T.; Banerjee, R., Construction of Crystalline 2D Covalent Organic Frameworks with Remarkable Chemical (Acid/Base) Stability via a Combined Reversible and Irreversible Route. *J. Am. Chem. Soc.* **2012**, 134, 19524-19527.
4. Zhang, Y.B.; Su, J.; Furukawa, H.; Yun, Y.; Gandara, F.; Duong, A.; Zou, X.; Yaghi, O.M., Single-crystal structure of a covalent organic framework. *J. Am. Chem. Soc.* **2013**, 135, 16336-16339.
5. Zhou, T.Y.; Xu, S.Q.; Wen, Q.; Pang, Z.F.; Zhao, X., One-Step Construction of Two Different Kinds of Pores in a 2D Covalent Organic Framework. *J. Am. Chem. Soc.* **2014**, 136, 15885-15888.
6. Wang, P.; Xu, Q.; Li, Z.P.; Jiang, W.M.; Jiang, Q.H.; Jiang, D.L., Exceptional Iodine Capture in 2D Covalent Organic Frameworks. *Adv. Mater.* **2018**, 30, 1801991.
7. Lu, Q.Y.; Ma, Y.C.; Li, H.; Guan, X.Y.; Yusran, Y.; Xue, M.; Fang, Q.R.; Yan, Y.S.; Qiu, S.L.; Valtchev, V., Postsynthetic Functionalization of Three-Dimensional Covalent Organic Frameworks for Selective Extraction of Lanthanide Ions. *Angew. Chem. Int. Ed.* **2018**, 57, 6042-6048.
8. Gao, C.; Li, J.; Yin, S.; Lin, G.; Ma, T.; Meng, Y.; Sun, J.; Wang, C., Isostructural Three-Dimensional Covalent Organic Frameworks. *Angew. Chem. Int. Ed.* **2019**, 58, 9770-9775.
9. Liu, X.L.; Li, J.; Gui, B.; Lin, G.Q.; Fu, Q.; Yin, S.; Liu, X.F.; Sun, J.L.; Wang, C., A Crystalline Three-Dimensional Covalent Organic Framework with Flexible Building Blocks. *J. Am. Chem. Soc.* **2021**, 143, 2123-2129.
10. Xie, Y.; Li, J.; Lin, C.; Gui, B.; Ji, C.; Yuan, D.; Sun, J.; Wang, C., Tuning the Topology of Three-Dimensional Covalent Organic Frameworks via Steric Control: From pts to Unprecedented ljh. *J. Am. Chem. Soc.* **2021**, 143, 7279-7284.
11. Sun, J.H.; Dong, S.Y.; Wang, Y.K.; Sun, S.P., Preparation and photocatalytic property of a novel dumbbell-shaped ZnO microcrystal photocatalyst. *J. Hazard. Mater.* **2009**, 172, 1520-1526.
12. Song, C.Y.; Yu, W.J.; Zhao, B.; Zhang, H.L.; Tang, C.J.; Sun, K.Q.; Wu, X.C.; Dong, L.; Chen, Y., Efficient fabrication and photocatalytic properties of TiO_2 hollow spheres. *Catal. Commun.* **2009**, 10, 650-654.
13. Yu, C.L.; Cao, F.F.; Li, X.; Li, G.; Xie, Y.; Yu, J.C.; Shu, Q.; Fan, Q.Z.; Chen, J.C., Hydrothermal synthesis and characterization of novel $PbWO_4$ microspheres with hierarchical nanostructures and enhanced photocatalytic performance in dye degradation. *Chem. Eng. J.* **2013**, 219, 86-95.
14. Du, J.J.; Yuan, Y.P.; Sun, J.X.; Peng, F.M.; Jiang, X.; Qiu, L.G.; Xie, A.J.; Shen, Y.H.; Zhu, J.F., New photocatalysts based on MIL-53 metal-organic frameworks for the decolorization of methylene blue dye. *J. Hazard. Mater.* **2011**, 190, 945-951.

15. Xu, N.; Wang, R.L.; Li, D.P.; Meng, X.; Mu, J.L.; Zhou, Z.Y.; Su, Z.M., A new triazine-based covalent organic polymer for efficient photodegradation of both acidic and basic dyes under visible light. *Dalton Trans.* **2018**, 47, 4191-4197.
16. Xie, J.; Jiang, D.; Chen, M.; Li, D.; Zhu, J.; Lü, X.; Yan, C., Preparation and characterization of monodisperse Ce-doped TiO_2 microspheres with visible light photocatalytic activity. *Colloid Surf. A-Physicochem. Eng. Asp.* **2010**, 372, 107-114.
17. Zhang, Y.M.; Hu, Y.M.; Zhao, J.H.; Park, E.; Jin, Y.H.; Liu, Q.J.; Zhang, W., Covalent organic framework-supported Fe-TiO_2 nanoparticles as ambient-light-active photocatalysts. *J. Mater. Chem. A* **2019**, 7, 16364-16371.
18. Wang, S.; Li, D.; Sun, C.; Yang, S.; Guan, Y.; He, H., Synthesis and characterization of g-C_3N_4/Ag_3VO_4 composites with significantly enhanced visible-light photocatalytic activity for triphenylmethane dye degradation. *Appl. Catal. B-Environ.* **2014**, 144, 885-892.
19. Cai, J.Y.; Liu, W.J.; Li, Z.H., One-pot self-assembly of Cu_2O/RGO composite aerogel for aqueous photocatalysis. *Appl. Surf. Sci.* **2015**, 358, 146-151.
